# Replication Unreliability in Psychology: Elusive Phenomena or “Elusive” Statistical Power?

**DOI:** 10.3389/fpsyg.2012.00218

**Published:** 2012-07-04

**Authors:** Patrizio E. Tressoldi

**Affiliations:** ^1^Dipartimento di Psicologia Generale, Università di PadovaPadova, Italy

**Keywords:** incubation effect, non-local perception, power, subliminal priming, unconscious thought theory

## Abstract

The focus of this paper is to analyze whether the unreliability of results related to certain controversial psychological phenomena may be a consequence of their low statistical power. Applying the Null Hypothesis Statistical Testing (NHST), still the widest used statistical approach, unreliability derives from the failure to refute the null hypothesis, in particular when exact or quasi-exact replications of experiments are carried out. Taking as example the results of meta-analyses related to four different controversial phenomena, subliminal semantic priming, incubation effect for problem solving, unconscious thought theory, and non-local perception, it was found that, except for semantic priming on categorization, the statistical power to detect the expected effect size (ES) of the typical study, is low or very low. The low power in most studies undermines the use of NHST to study phenomena with moderate or low ESs. We conclude by providing some suggestions on how to increase the statistical power or use different statistical approaches to help discriminate whether the results obtained may or may not be used to support or to refute the reality of a phenomenon with small ES.

## Introduction

### Are there elusive phenomena or is there an “elusive” power to detect them?

When may a phenomenon be considered to be real or very probable, following the rules of current scientific methodology? Among the many requirements, there is a substantial consensus that replication is one of the more fundamental (Schmidt, 2009). In other words, a phenomenon may be considered real or very probable when it has been observed many times and preferably by different people or research groups. Whereas a failure to replicate is quite expected in the case of conceptual replication, or when the experimental procedure or materials entail relevant modifications, a failure in the case of an exact or quasi-exact replication, give rise to serious concerns about the reality of the phenomenon under investigation. This is the case in the four phenomena used as examples in this paper, namely, semantic subliminal priming, incubation effects on problem solving, unconscious thought, and non-local perception (NLP; e.g., Kennedy, 2001; Pratte and Rouder, 2009; Waroquier et al., 2009).

The focus of this paper is to demonstrate that for all phenomena with a moderate or small effect size (ES), approximately below 0.5 if we refer to standardized differences such as Cohen’s *d*, the typical study shows a power level insufficient to detect the phenomenon under investigation.

Given that the majority of statistical analyses are based on the Null Hypothesis Statistical Testing (NHST) frequentist approach, their failure is determined by the rejection of the (nil) null hypothesis H_0_, usually setting α < 0.05. Even if this procedure is considered incorrect because the frequentist approach only supports H_0_ rejection and not H_0_ validity^1^, it may be tolerated if there is proof of a high level of statistical power as recommended in the recent APA statistical recommendations [American Psychological Association, APA (2010)]: “Power: When applying inferential statistics, take seriously the statistical power considerations associated with the tests of hypotheses. Such considerations relate to the likelihood of correctly rejecting the tested hypotheses, given a particular alpha level, ES, and sample size. In that regard, routinely provide evidence that the study has sufficient power to detect effects of substantive interest. Be similarly careful in discussing the role played by sample size in cases in which not rejecting the null hypothesis is desirable (i.e., when one wishes to argue that there are no differences), when testing various assumptions underlying the statistical model adopted (e.g., normality, homogeneity of variance, homogeneity of regression), and in model fitting. pag. 30.”

### How much power?

Statistical power depends on three classes of parameters: (1) the significance level (i.e., the Type I error probability) of the test, (2) the size(s) of the sample(s) used for the test, and (3) an ES parameter defining H_1_ and thus indexing the degree of deviation from H_0_ in the underlying population.

Power analysis should be used prospectively to calculate the minimum sample size required so that one can reasonably detect an effect of a given size. Power analysis can also be used to calculate the minimum ES that is likely to be detected in a study using a given sample size.

In most experimental designs, the accepted probability of making a Type I error is α = 0.05 and the desired power is not less than 0.80. However, in order to define how to obtain such a level of power, it is necessary to know the ES of the phenomena being identified. It is intuitive that the smaller the phenomenon, the greater should be the means to detect it. This analogy is similar to the signal/noise relationship. The smaller the signal, the stronger must be the means to detect it in the noise. In psychological experiments, these means are the number of participants taking part in the study and the number of trials they are requested to perform.

Given that Power = 1−β = ES* ✓N/SD* α, if we know the estimated ES of a phenomenon, after the definition of the desired power and the α level, the only free parameters is *N*, that is the number of participants or trials.

A review of 322 meta-analyses published before 1998, summarizing 25,000 studies referred to 474 social psychological effects, reports that the mean ES reported is *r* = 0.21 and the mode was less than *r* = 0.10 (Richard et al., 2003). For this observed mean ES, to obtain a statistical power for independent sample *t* tests = >0.9, the sample size for each group should be at least 90, a number rarely observed in the studies.

Setting aside the strong criticisms of the use of NHST (Cohen, 1994; Kline, 2004), a neglected aspect of this approach, is the control of how much statistical power is necessary to detect what the researcher aims to find^2^.

This problem is not new and has already been raised by Sedlmeier and Gigerenzer (1989), Cohen (1992), Bezeau and Graves (2001), and Maxwell (2004) among others. However the widespread adherence to “The Null Ritual” as discussed by Gigerenzer et al. (2004), which consists in: (a) Set up a statistical null hypothesis of “no mean difference” or “zero correlation.” (b) Don’t specify the predictions of your research hypothesis or of any alternative substantive hypotheses; (c) Use 5% as a convention for rejecting the null; (d) If significant, accept your research hypothesis (e) Always perform this procedure, seems to prevent most researchers taking into account such fundamental statistical parameter. In Gigerenzer et al. (2004) check, covering the years 2000–2002, and encompassing with some 220 empirical articles, only nine researchers who computed the power of their tests were found.

Using the results of four recent meta-analyses related to “controversial” or “elusive” psychological phenomena, we illustrate the importance of using the available ESs to derive the appropriate number of participants to achieve a power = >0.90. Only if a replication fails with this level of power, it is legitimate to raise doubts about the reality of the phenomena under investigation.

### Can subliminally presented information influence behavior?

This question is one of the most controversial questions in psychology and it remains an intriguing and strongly debated issue. Apart from the controversies relating to the controls that information (priming) was effectively masked and the identification of serious methodological flaws which caused great doubt as to the existence of subliminal processing, the debate is even hotter around the topic of the level of influence of this unconscious (subliminal) information. Hitherto, the debate as to whether subliminal priming reflects genuine semantic processing of the subliminal information or the formation of automatic S–R mappings remains unresolved.

The meta-analysis of Van den Bussche et al. (2009) tried to shed light on these questions by analyzing all the available literature between 1983 and December 2006. Their analysis was carried out separately from the two most studied protocols, subliminal priming for semantic categorization and subliminal priming for lexical decision and naming. If semantic subliminal priming facilitated the subsequent categorization of targets belonging to the same semantic category, it suggests that the primes were unconsciously categorized and processed semantically. The same effect is postulated if lexical decision and naming are faster or more accurate to semantically subliminal related prime–target pairs than to unrelated pairs. A naming task is similar to the lexical decision task except that the targets are all words, and participants are asked to name the targets aloud.

The synthesis of the main results is reported in Table 1.

**Table 1:** **Descriptive statistics of the four meta-analyses, related to Unconscious Semantic Priming, Incubation effect, UTT, and NLP with the estimated power of a typical study, the mean and 95% CI power calculated from all studies included in the meta-analysis and the number of participants necessary to obtain a Power = 0.90**.

Phenomena	Protocols	Source	*N* studies	Averaged *N* × study (range)	ES* (mean and 95% CI)	*p*	Estimated power of a typical study	Observed power (mean and 95% CI)	Estimated **N** to achieve power = 0.90
Semantic priming	Semantic categorization	Van den Bussche et al. (2009)	23	20 (6–80)	0.80 ± 0.2	n.a.	0.96	0.90 (±1.7)	
	Lexical and naming	Van den Bussche et al. (2009)	32	33 (9–132)	0.47 ± 0.11	n.a.	0.84	0.69 (±0.4.6)	40
Incubation effect		Sio and Ormerod (2009)	117	31 (7–278)	0.29 ± 0.09	n.a.	0.21	0.43 (±3.3)	100
Unconscious thought theory		Strick et al. (2011)	92	26 (14–55)	0.22 ± 0.08	1.2 × 10^−8^	0.20§	0.19 (±0.72)	400§
Non-local perception	Remote Vision	Milton (1997)	78	34 (1–74)	0.16 ± 0.06	6 × 10^−9^	0.55	n.a.	73
	Ganzfeld	Storm et al. (2010)	108	40 (7–128)	0.13 ± 0.04	8.3 × 10^−11^	0.46	0.45 (±3.8)	110
	Forced-choice with normal state of consciousness	Storm et al. (in press)	72	128 (12–887)	0.011 ± 0.004	5.3 × 10^−7^	0.07	0.065 (±0.81)	3450

*Random effect Cohen’s *d*; § two groups comparison.

### Does incubation enhance problem solving?

The “incubation period” is the temporary shift away from an unsolved problem in order to allow a solution to emerge in the mind of the individual, seemingly with no additional effort, after he or she has put the problem aside for a period of time, having failed in initial attempts to solve it.

Among the questions under debate there is the problem of whether the nature of the discovery of the solution is really unconscious and if it is qualitatively different from that used to tackle problems that do not require such insight.

The meta-analysis of Sio and Ormerod (2009) tried to shed light on this and other related questions, analyzing all the available literature from 1964 to 2007. The main findings of their meta-analysis are reported in Table 1.

### Unconscious thought theory

The key assumption of Unconscious Thought Theory (UTT, Dijksterhuis et al., 2006) is that unconscious thought and conscious thought are characterized by different processes. That is, “unconscious thought” processes have a relatively large capacity – hence, they allow for an optimal decision strategy in which all attributes of chosen alternatives are weighted according to their importance. These unconscious processes require time, therefore the quality of decisions increases with the duration of unconscious thought. “Conscious thought” processes on the other hand, have a small capacity and therefore only allow for simplified decision making strategies. As summarized by Dijksterhuis et al., 2006, p. 105): “When a decision strategy warrants the careful and strict application of one specific rule, as in a lexicographic strategy, use conscious thought. When matters become more complicated and weighting is called for, as in the weighting strategy, use unconscious thought.”

As expected, among the questions raised by this theory, a critical problem is whether really optimal decisions are obtained after a period of distraction from deliberate conscious mental activity for the same amount of time as would be the case had the decisions been taken deliberately.

The meta-analysis of Strick et al. (2011), aimed to give an answer to this and other related questions by analyzing all the available evidence up to May 2011. The main findings are reported in Table 1.

### Non-local Perception

Non-local perception (NLP) is based on the hypothesis that the human mind may have quantum like properties, that is, that some of its functions, such as perceptual abilities, reasoning, etc., may be analyzed using quantum formalism.

The main non-local properties which are studied within the realm of quantum physics and which are supported by “extraordinary evidence” (see Genovese, 2005, 2010), are “entanglement” and “measurement interference.” The first property, entanglement, allows two or more physical objects to behave as one even if they are separated in space and time. This “strange” property allows a form of immediate communication of the objects’ characteristics over distances between or among the entangled objects, as has been observed in teleportation experiments (i.e., Bouwmeester et al., 1997). The possibility that quantum-like properties may be observed not only in physics but also even in biology and psychology has not only been studied theoretically (von Lucadou et al., 2007; Khrennikov, 2010; Walach and von Stillfried, 2011) but also experimentally (see Gutiérrez et al., 2010 for biology and Busemeyer et al., 2011, for psychology).

One of the main concerns about the studies related to different aspects of NLP, is their inconsistency in obtaining results satisfying the statistical cut off criteria to refute the null hypothesis that extra sensory perception does not exist, usually setting α < 0.05.

This problem is recognized by some researchers involved in this field of research (Kennedy, 2001) and even more by all deniers of NLP evidence (e.g., Alcock, 2003).

The main findings of three meta-analysis related to three NLP different protocols, Remote Vision, that is NLP using a free-choice response (Milton, 1997), NLP in a Ganzfeld state using free-choice response (Storm et al., 2010) and NLP in a normal state of consciousness using a forced-choice response (Storm et al., in press), covering all evidence available from 1964 to 1992, 1974 to 2009, and 1987 to 2010 respectively, are reported in Table 1

### Power estimation

A synthesis of the descriptive statistics related to the four phenomena described above is presented in Table 1 in decreasing order of magnitude of ESs. For each meta-analysis, the retrospective statistical power with α = 0.05, achieved by a typical study using the mean of the number of participants of all studies included in the meta-analysis was estimated.

For all but one meta-analysis, it was also possible to calculate the mean and 95% CI *post hoc* power, using the number of participants of each study included in the meta-analyses and the estimated random ES, setting α = 0.05.

Furthermore, for each of the four psychological phenomena, the number of participants necessary to obtain a statistical power = 0.9 with α = 0.05 given the observed random ESs, was estimated.

Statistical power was calculated using the software G*Power (Faul et al., 2007).

### Comment

The results are quite clear: apart from the unconscious semantic priming for semantic categorization, where the number of participants in a typical experiment is sufficient to obtain a statistical power above 0.90, for all remaining phenomena, to achieve this level of power, it is necessary to increase the number of participants in a typical study, from a minimum of seven participants for the unconscious semantic priming for lexical decision and naming to around 3400 to investigate NLP using the forced-choice with normal state of consciousness protocol.

## General Discussion

The response to the question posed in the introduction, as to whether there are elusive phenomena or an elusive power to detect them, is quite clear. If there are clear estimates of ESs from the evidence of the phenomenon derived from a sufficient number of studies analyzed meta-analytically and their values are moderate or low, it is mandatory to increase the number of participants to achieve a statistical power of 0.90, with the inevitable consequence of investing more time and money into each study before interpreting the results as support for reality or unreality of a phenomenon.

Are there alternatives to this obligation? Yes, and we briefly illustrate some of these, also providing references for those interested in using them.

### Confidence intervals

In line with the statistical reform movement (i.e., Cumming, 2012), in the APA manual (American Psychological Association, APA, 2010), there are the following statistical recommendations “Alternatively, (to the use of NHST) use calculations based on a chosen target precision (confidence interval width) to determine sample sizes. Use the resulting confidence intervals to justify conclusions concerning ESs (e.g., that some effect is negligibly small) p. 30.”

### Equivalence testing

Equivalence tests are inferential statistics designed to provide evidence for a null hypothesis. Like effect tests, the nil–null is eschewed in equivalence testing. However unlike standard NHST, equivalence tests provide evidence that there is little difference or effect. A significant result in an equivalence test means that the hypothesis that the effects or differences are substantial can be rejected. Hence, equivalence tests are appropriate when researchers want to show little difference or effect (Levine et al., 2008).

### Evaluating informative hypotheses

Evaluating specific expectations directly produces more useful results than sequentially testing traditional null hypotheses against catch-all rivals. Researchers are often interested in the evaluation of informative hypotheses and already know that the traditional null hypothesis is an unrealistic hypothesis. This presupposes that prior knowledge is often available; if this is not the case, testing the traditional null hypothesis is appropriate. In most applied studies, however, prior knowledge is indeed available in the form of specific expectations about the ordering of statistical parameters (Kuiper and Hoijtink, 2010; Van de Schoot et al., 2011).

### Bayesian approach

Another alternative is to abandon the frequentist approach and use a Bayesian one (Wagenmakers et al., 2011). With a Bayesian approach the problem of statistical power is substituted with parameter estimation and/or model comparison (Kruschke, 2011). In the first approach, assessing null values, the analyst simply sets up a range of candidate values, including the null value, and uses Bayesian inference to compute the relative credibility of all the candidate values. In the model comparison approach, the analyst sets up two competing models of what values are possible. One model posits that only the null value is possible whereas the alternative model posits that a broad range of other values is also possible. Bayesian inference is used to compute which model is more credible, given the data.

## Final Comment

Is there a chance to abandon “The Null Ritual” in the near future and to think of science as cumulative knowledge? The answer is “yes” if we approach scientific discovery thinking meta-analytically (Cumming, 2012), that is, simply reporting observed (standardized) ES and the corresponding confidence intervals, both when NHST is refuted and when it is not refuted (Nickerson, 2000; American Psychological Association, APA, 2010) without drawing dichotomous decisions. The statistical approaches listed above are good tools to achieve this goal.

How many editors and reviewers are committed to pursuing it?

## Conflict of Interest Statement

The author declares that the research was conducted in the absence of any commercial or financial relationships that could be construed as a potential conflict of interest.

## Footnotes

^1^The line of reasoning from “the null hypothesis is false” to “the theory is therefore true” involves the logical fallacy of affirming the consequent: “If the theory is true, the null hypothesis will prove to be false. The null hypothesis proved to be false; therefore, the theory must be true” (Nickerson, 2000).

^2^Even if power estimate is important in meta-analyses (i.e., Valentine et al., 2009), in this paper we focus only on power estimates in single studies.
